# Nanoencapsulated senotherapeutic compounds targeting connexin-43 for enhanced wound healing

**DOI:** 10.1016/j.isci.2025.114547

**Published:** 2025-12-26

**Authors:** Marina Rodríguez-Candela Mateos, Jenifer García-Fernández, Sofia M. Saraiva, Maria Aurora Grimaudo, Sandra Alijas, Adela Escudero, Marta Varela-Eirín, Juan Pérez Cano, Benigno Acea Nebril, Luis C. Barrio, María D. Mayan, María de la Fuente

**Affiliations:** 1 Institute of Biomedical Research of A Coruña (INIBIC), Complexo Hospitalario Universitario de A Coruña (CHUAC), SERGAS, A Coruña 15006, Spain; 2 Nano-Oncology and Translational Therapeutics Group, Health Research Institute of Santiago de Compostela (IDIS), Clinical University Hospital of Santiago de Compostela (CHUS), SERGAS, Santiago de Compostela 15706, Spain; 3 Unit of Experimental Neurology, “Ramón y Cajal” Hospital (IRYCIS), Madrid 28034, Spain; 4 Alodia Farmacéutica SL, Santiago Grisolía 2 D130/L145, Madrid 28108, Spain; 5 Surgery Department, Breast Unit, INIBIC, A Coruña University Hospital, SERGAS, A Coruña 15004, Spain; 6 DIVERSA Technologies SL, Santiago de Compostela 15782, Spain; 7 Cancer Network Research (CIBERONC), Madrid 28029, Spain

**Keywords:** biological sciences, engineering, materials science

## Abstract

Oncological patients receiving radiotherapy or chemotherapy often experience impaired wound healing due to tissue damage and cellular senescence. Connexin43 (Cx43) is a key regulator in this process, promoting senescence and the SASP, which hinder regeneration. This study investigates targeting Cx43 in primary dermal fibroblasts from cancer patients using a new combination of nanoencapsulated drugs. We show that Cx43 overexpression correlates with reduced gap junction communication, increased senescence, and delayed healing. Treatment with oleuropein (OLP), a polyphenol with antioxidant and regenerative properties, restores fibroblast function, reduces senescence, and improves healing. We also examine the p38 MAPK inhibitor BIRB796, alone and with OLP, demonstrating its ability to suppress SASP and support tissue repair. After confirming the superior combined effect of OLP and BIRB796, we developed a co-encapsulated nanoformulation. This innovative approach may significantly enhance wound healing in oncological patients.

## Highlights

• Cx43 alterations impairs healing by promoting senescence in cancer patient skin fibroblasts
• Oleuropein restores fibroblast function and reduces cellular senescence
• BIRB796 reduces SASP in senescent cancer patient fibroblasts, promoting regenerative repair
• Nano-coencapsulated OLP+BIRB796 enhances delivery and wound-healing efficacy

## Graphical abstract

**Figure:**
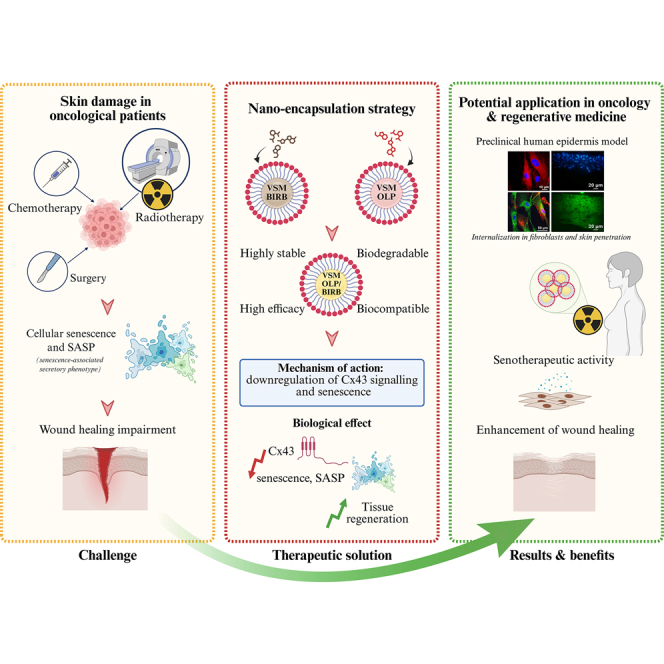

## Introduction

The impairment in wound healing of oncological patients, particularly due to radiotherapy and chemotherapy, is a well-documented challenge with few treatment options.^1^ Radiotherapy, often used in combination with surgery and chemotherapy, involves administering high-energy ionizing radiation to halt malignant tumor growth. However, it also damages healthy tissues, including skin, affecting crucial cell types for healing, such as macrophages and fibroblasts.^2^^,^^3^ The consequences of radiation injury to the skin can be severe and long-lasting. Patients may experience cutaneous atrophy, soft tissue fibrosis and microvascular damage, leading to a higher risk of non-healing acute wounds.^4^ Of note, the range of available treatment approaches for alleviating radiation and chemotherapeutic-induced skin damage is limited.^5^^,^^6^

Cellular senescence, a state of cell-cycle arrest, is characterized by sustained metabolic activity and secretion of the Senescence Associated Secretory Phenotype (SASP).^7^^,^^8^ While transient senescence can be beneficial for proper wound healing, persistent accumulation of senescent cells in off-target tissues like skin can result in impaired healing and even organ dysfunction if not removed.^9^^,^^10^^,^^11^^,^^12^^,^^13^ Indeed, chronic senescence has been implicated in non-healing human ulcers induced by radiation^13^ and in diabetic murine slow-healing wounds.^14^ Similarly, a great number of chemotherapy agents currently used in the clinic are associated with senescence induction in tumors, but also in off-target tissues, such as the skin.^15^ Therapeutics targeting the activity of the most ubiquitous gap junction protein, connexin43 (Cx43), show promise in beneficially modulating the human body’s natural healing response for improved patient outcomes across a variety of injuries.^16^ Downregulation of Cx43 activity or protein levels has demonstrated a decrease in senescence and its inflammatory phenotype and an increase in tissue regeneration.^17^^,^^18^

To this end, nanotechnology emerges as a highly promising approach to overcome the limitations of conventional wound therapies. Nanomaterials offer multifunctional solutions, addressing key challenges in wound management such as infection control, inflammation management, and tissue regeneration.^19^^,^^20^ Nanoparticles, in particular, have demonstrated utility in the encapsulation and delivery of polyphenols,^21^ as well as hydrophobic and hydrophilic drugs, for targeted skin delivery.^22^ According to the literature, small-sized nanoparticles present a higher capacity to permeate through the skin.^23^ To design an optimal formulation for skin delivery, careful selection of materials is essential to ensure efficient encapsulation, skin interaction, and controlled release.

In this study, we propose the use of biocompatible nanoemulsions composed of natural, biodegradable sphingomyelin (SM) and vitamin E (VitE), VSM-based nanoemulsions, which were previously developed by our group.^24^ SM, a major lipid in cell membranes,^25^ plays a critical role in skin barrier function, hydration, and protection. Its conversion to ceramides and interaction with other skin lipids make it a key player in maintaining skin health and addressing various dermatological concerns.^26^ In addition, SM has demonstrated efficacy in improving skin barrier function, as shown in studies involving dogs with atopic dermatitis.^27^ Vitamin E, on the other hand, is a well-established antioxidant and photoprotective agent,^28^^,^^29^ validated for various nanoformulations, including delivery of small molecules, biomolecules, and peptides capable of modulating senescence.^30^^,^^31^^,^^32^^,^^33^ The incorporation of GRAS (generally recognized as safe) materials such as SM and VitE ensures biocompatibility and safety for clinical use of the developed nanoemulsions. VitE serves as the oily core for drug encapsulation, while SM acts as a stabilizer.^24^ VSM-based nanoemulsions can be prepared using low-energy methods, which preserve the stability of the associated molecules,^24^^,^^31^ and have been previously validated for the delivery of hydrophilic and lipophilic drugs, showing enhanced drug stability, bioavailability, and rapid cellular uptake both *in vitro* and *in vivo.*^30^^,^^31^^,^^32^^,^^33^^,^^34^^,^^35^^,^^36^^,^^37^

To further enhance the permeability and therapeutic performance of these nanosystems, we optimized their composition by incorporating surfactants such as D-α-tocopherol polyethylene glycol 1000 succinate (TPGS), sodium deoxycholate (SDC), and sodium taurocholate (STC). TPGS, a water-soluble derivative of VitE approved by the FDA as a formulation adjuvant, has been widely used to improve drug oral bioavailability^38^ and enhance tissue penetration, including the skin^39^^,^^40^ and cornea.^41^ In addition, TPGS demonstrates pro-apoptotic effects against various cancer cell types.^42^^,^^43^ Complementing this, bile acids such as SDC and STC, were integrated for their well-known penetration-enhancing properties. These bile acids have been successfully employed in commercially available micelle-based formulations for parenteral drug delivery^44^ and the development of skin-targeted nanocarriers.^45^^,^^46^ Furthermore, bile acids, including glycolic acid derivatives, are commonly utilized in skin care applications.

The proposed nanoemulsion-based senotherapeutic systems offer several advantages for skin treatments, including high encapsulation efficiency (EE), sustained drug release for prolonged therapeutic effects, and enhanced intracellular uptake. Importantly, VSM-nanoemulsions can synergize with oleuropein (OLP), a natural polyphenol with proven wound-healing properties,^47^^,^^48^ and the p38 inhibitor BIRB796. By leveraging these components, our approach aims to improve post-radiation wound healing outcomes by modulating Cx43 expression and its role in SASP, particularly in oncology patients. These findings collectively underscore the clinical potential of our nanosystems for improving skin regeneration and addressing the persistent wound healing challenges faced by patients with skin damage caused by chemotherapy and radiotherapy.

## Results

### Assessment of Cx43 expression and GJIC in dermal fibroblasts from breast cancer patients subjected to chemo- and radiotherapy

**Figure 1:**
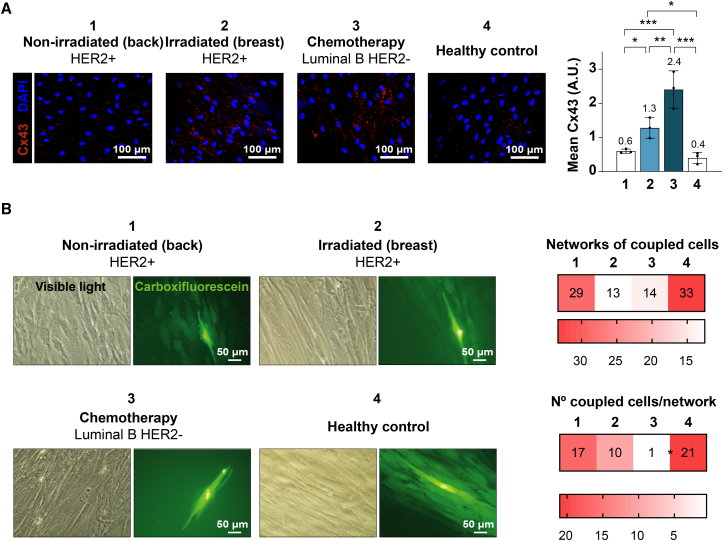
Cx43 is overexpressed but GJIC is impaired in dermal fibroblasts isolated from the skin of breast cancer patients subjected to chemotherapy and radiotherapy (A) Representative immunofluorescences against Cx43 (red) in dermal fibroblasts isolated from the untreated (1 and 4) and chemo- (3) and radiotherapy (2)-treated skin of breast cancer patients. Nuclei are stained with DAPI (blue). Graph represents mean Cx43 red fluorescence relativized to cell density. Mean ± SD; mean values specified in the graph. *n* = 3 pictures/patient, each *n* is represented as a dot in the graph. One-way ANOVA. ∗*p* < 0.05, ∗∗*p* < 0.01, ∗∗∗*p* < 0.001. Scale bars = 100 μm. (B) Representative visible light and corresponding fluorescence images of dye-coupling experiments in fibroblasts isolated from untreated (1 and 4) and chemo- (3) and radiotherapy (2)-treated skin of breast cancer patients. Injected 5(6)-carboxyfluorescein is shown in bright green, and dye transferred to neighboring cells by gap junctions appears in lighter green. Assay quantitation is shown as the networks of coupled cells (defined as the percentage of carboxyfluorescein-injected cells found coupled to at least one adjacent cell) and the number of coupled cells per network. Mean values are specified in the heatmaps. *t* test. ∗*p* < 0.05. Scale bars = 50 μm.

First, Cx43 expression was assessed by immunofluorescence in dermal fibroblasts isolated from the skin of untreated (1 and 4) and chemo- (3) and radiotherapy-treated (2) breast cancer patients (Figure 1A). Samples 1 and 2 belong to different areas of the same patient (HER2+ subtype); the former is from the untreated back region, and the latter is from the radiotherapy-treated breast. Sample 3 is from a luminal B HER2 patient who had undergone neoadjuvant chemotherapy 6 weeks prior; sample 4 is derived from a patient with benign pathology undergoing mammoplasty. Cx43 was found to be upregulated in all samples compared to non-irradiated or untreated control skin (Figure 1A). Next, gap junction functionality was assessed in these fibroblasts by dye coupling assays, in which 5(6)-carboxyfluorescein gap junction-permeable dye (green) was injected in one cell of a monolayer and its transfer was evaluated as GJIC (Figure 1B). Strikingly, even though fibroblasts isolated from the skin of chemo- and radiotherapy-treated patients (samples 2 and 3) showed higher Cx43 levels (Figure 1A), their gap junction functionality seemed to be impaired when compared to untreated fibroblasts (samples 1 and 4) (Figure 1B).

### Evaluation of senomodulating compounds on Cx43 expression and GJIC in fibroblasts exposed to chemo- and radiotherapy

**Figure 2:**
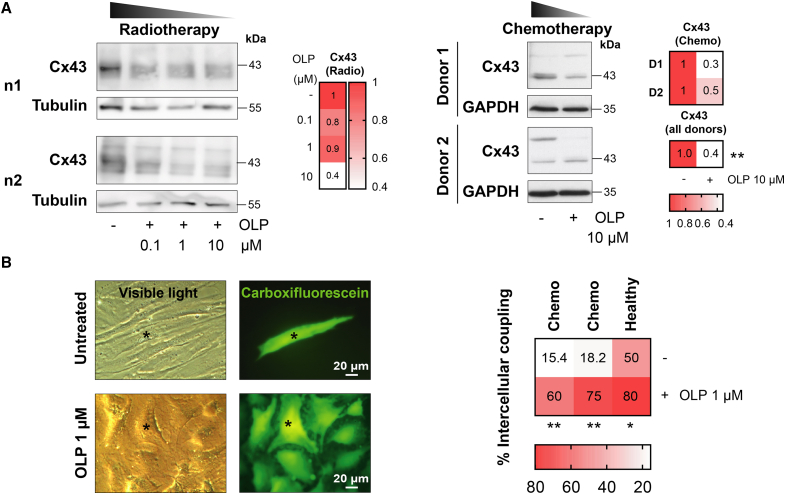
OLP treatment reduces Cx43 levels and restores the GJIC of dermal fibroblasts isolated from the skin of breast cancer patients exposed to chemotherapy and radiotherapy (A) Representative western blots against Cx43 in dermal fibroblasts isolated from the skin of breast cancer patients subjected to radiotherapy (left, 1 donor) or chemotherapy (right, 2 donors), treated with various concentrations (0.1, 1, and 10 μM) of OLP for 2 h. Tubulin and GAPDH are used as loading controls. Mean values specified in the heatmaps. *t* test. ∗∗*p* < 0.01. (B) Quantitation of the percentage of intercellular coupling and representative images of dye coupling experiments in fibroblasts isolated from breast cancer patients exposed or not to chemotherapy, treated with OLP 1 μM for 4 h 5(6)-carboxyfluorescein-injected cell (green) is marked with an asterisk. Mean values are specified in the heatmap. *n* = 2–3. *t* test. ∗*p* < 0.05, ∗∗*p* < 0.01. Scale bars = 20 μm.

In order to address the previously detected Cx43-GJIC dysfunction in radio- and chemotherapy-treated fibroblasts (Figure 1), several senomodulating synthetic and natural compounds were tested (data not shown), namely quercetin, dasatinib, navitoclax and OLP. Among these components, it was found that naturally occurring polyphenol OLP, particularly enriched in olive oil and the leaves of olive trees,^49^ reduced Cx43 elevated levels in fibroblasts isolated from the skin of radio- (left) and chemotherapy-treated (right) patients in a concentration-dependent manner (0.1, 1, and 10 μM, 2 h) (Figure 2A). Additionally, it also improved the functionality of the already-impaired GJIC in chemotherapy-exposed as well as in healthy fibroblasts (preliminary test assay with 1 μM 4 h), as assessed by dye coupling experiments (5(6)-carboxyfluorescein-injected cell marked with an asterisk). Interestingly, OLP treatment also induced a shift in the cellular phenotype of chemotherapy-treated fibroblasts toward a more mesenchymal-like phenotype, which is crucial for the wound healing process (Figure 2B). The 10 μM OLP concentration was chosen for all subsequent assays based on the consistent reduction in Cx43 expression observed at this dose (Figure 2A), as well as prior evidence from our group and others supporting this concentration in terms of Cx43/senescence modulation and cell differentiation.^18^^,^^50^

### Mechanistic insights into senescence and SASP modulation in irradiated fibroblasts and pro-healing effects of combined OLP-BIRB796 treatment

**Figure 3:**
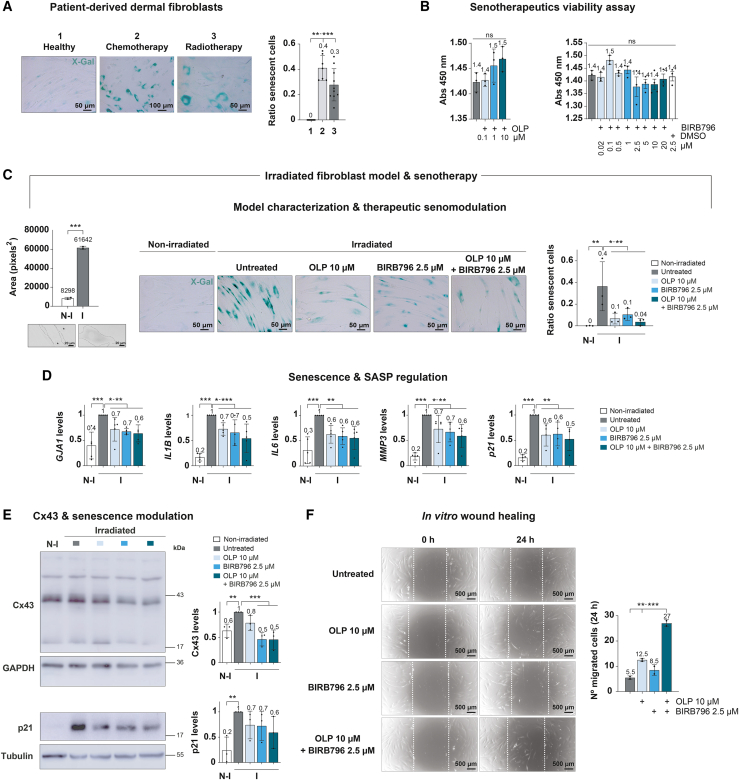
Free OLP and BIRB796 treatment effect on senescence modulation and pro-healing in experimentally irradiated dermal fibroblasts (A) Representative images of the detection of beta-galactosidase (SA-βgal) activity by X-Gal staining (blue) in fibroblasts isolated from the skin of untreated (1), chemo- (2) and radiotherapy (3)-treated breast cancer patients. Graph represents the senescent cells ratio per image field analyzed. Mean ± SD; mean values specified in the graph. *n* = 7–10 images/donor, each image measurement is represented as a dot in the graph. *t* test. ∗∗*p* < 0.01, ∗∗∗*p* < 0.001, ns = not significant. Scale bars = 50, 100 μm. (B) Proliferation assay of experimentally irradiated (55 Gy) dermal fibroblasts treated with various concentrations of OLP (0.1, 1, and 10 μM) or BIRB796 (0.02, 0.1, 0.5, 1, 2.5, 5, 10, 20 μM, and DMSO treatment corresponding to the chosen 2.5 μM dose) for 72 h. Mean ± SD; mean values specified in the graph. *n* = 3–4, each *n* is represented as a dot in the graph. One-way ANOVA. ns = not significant. (C) (Left) Differential phenotypic characterization of healthy and experimentally irradiated (55 Gy) dermal fibroblasts. Scale bars = 50 μm. Graph represents the average cellular area in each image field analyzed. Mean ± SD; mean values specified in the graph. *n* = 2 (4 images/*n*, 30–45 cells/*n*), each *n* measurement is represented as a dot in the graph. *t* test. ∗∗∗*p* < 0.001. The brightfield images below show representative cells from each condition, with their contours outlined by dashed lines; asterisks (∗) denote rounded cells consistent with mitotic figures. (Right) Representative images of the detection of beta-galactosidase (SA-βgal) activity by X-Gal staining (blue) in non-irradiated and experimentally irradiated (55 Gy) fibroblasts, treated with 10 μM OLP, 2.5 μM BIRB796, or their combination treatment for 72 h. Graph represents the senescent cells ratio per image field analyzed. Mean ± SD; mean values specified in the graph. *n* = 3 (5 images/*n*), each image measurement is represented as a dot in the graph. *t* test. ∗*p* < 0.05, ∗∗*p* < 0.01, ∗∗∗*p* < 0.001. (D) qPCR analysis of mRNA levels of *GJA1*, SASP factors (*IL1B* and *IL6*) and senescence-related components (*MMP3* and *p21*) in non-irradiated and experimentally irradiated (55 Gy) fibroblasts, treated with 10 μM OLP, 2.5 μM BIRB796, or their combination treatment for 72 h. All data were relativized to their respective *HPRT-1* expression, and then to that of irradiated untreated fibroblasts. Mean ± SD, mean values specified in the graph. *n* = 4–5, each *n* is represented as a dot in the graph. *t* test. ∗*p* < 0.05, ∗∗*p* < 0.01, ∗∗∗*p* < 0.001. (E) Representative western blots against Cx43 and p21 in non-irradiated and experimentally irradiated (55 Gy) fibroblasts, treated with 10 μM OLP, 2.5 μM BIRB796, or their combination treatment for 72 h. GAPDH and tubulin are used as loading controls. Data quantitation is normalized to their respective loading control expression, and then to that of irradiated untreated fibroblasts. Mean ± SD, mean values specified in the graph. *n* = 3, each *n* is represented as a dot in the graph. *t* test. ∗∗*p* < 0.01, ∗∗∗*p* < 0.001. (F) Representative 0 and 24-h pics of scratch/wound healing assay performed in experimentally irradiated (55 Gy) dermal fibroblasts treated with 10 μM OLP, 2.5 μM BIRB796, or their combination treatment for 24 h. The original scratched area is defined by dashed white lines. The quantitation graph shows the number of migrated cells to the scratched area after 24 h. Mean ± SD, mean values specified in the graph. *n* = 2, each *n* is represented as a dot in the graph. *t* test. ∗∗*p* < 0.01, ∗∗∗*p* < 0.001. N-I = non-irradiated. I = irradiated. Scale bars = 500 μm.

To further characterize the underlying mechanism of the wound healing impairment in oncological patients exposed to radiotherapy and chemotherapy, senescence-associated beta-galactosidase (SA-βgal) activity was evaluated by X-Gal staining (blue) (Figure 3A), and it was found that fibroblasts isolated from the skin of chemo- and radiotherapy-treated patients presented markedly higher senescence levels than healthy untreated ones (30%–40% senescent cells versus 0% senescent cells on average, respectively). These results were successfully recapitulated in experimentally irradiated (55 Gy) dermal fibroblasts (Figure 3C). This experimental irradiation managed to significantly increase overall senescence of previously healthy fibroblasts to a similar degree (40% senescent cells on average, assessed 7 days post-irradiation) (Figure 3C, right) as that observed in fibroblasts isolated from radio- and chemotherapy-treated patients (Figure 3A). SA-βgal activity remains a standard and widely used hallmark of senescence; however, more recent approaches combining Ki67, pRPS6, and SA-βgal allow a finer distinction between senescent, quiescent, and stressed cells.^51^ To strengthen our characterization of radiation-induced senescence and growth arrest in our available materials, we performed mitotic figure counting (defined as rounded, refractile morphology, and/or visible cleavage furrows), revealing a complete absence of mitoses in irradiated fibroblasts, compared to occasional mitoses in non-irradiated samples (Figure 3C, left). In line with this, the average cellular area was significantly enlarged in the experimentally irradiated fibroblasts compared to healthy ones (Figure 3C, left). These observations, together with the significant increase in the senescence marker p21 after irradiation (Figures 3D and 3E), are consistent with cell-cycle withdrawal upon irradiation. 10 μM OLP treatment for 72 h post-irradiation (after 4 days of recovery +72 h treatment) managed to significantly reduce senescent cell accumulation as determined by X-Gal staining (Figure 3C). To enhance OLP performance, it was combined with other molecules to reduce not only senescence but also the synthesis of SASP factors. Among the tested compounds, BIRB796 was selected due to the crucial role of the p38 MAPK pathway in promoting senescence and SASP in fibroblasts.^12^^,^^52^^,^^53^ The 2.5 μM working concentration was determined according to literature reports regarding its efficacy^54^ and not causing significant cytotoxicity on irradiated fibroblasts, as neither did the 10 μM OLP selected dose (Figure 3B). This OLP-BIRB796 combination treatment also significantly ameliorated the senescent status of irradiated fibroblasts, in a trend that suggested improvement over OLP treatment alone (Figure 3C).

The gene expression of several SASP factors (*IL1B* and *IL6*) and other senescence-related elements (*MMP3* and *p21*) was analyzed by RT-qPCR in experimentally irradiated (55 Gy) dermal fibroblasts (Figure 3D; Table 1). Experimental irradiation, as implied in the point before (Figure 3B), induced a steep increase in the expression of SASP and senescence factors, as well as in *GJA1*, which encodes Cx43. 10 μM OLP, 2.5 μM BIRB796, or their combination treatment for 72 h post-irradiation significantly reduced their levels, and the decline was more acute when both treatments were applied (Figure 3D). Furthermore, Cx43 and p21 overexpression in dermal fibroblasts after experimental irradiation (55 Gy) and reduction after OLP-BIRB796 treatment for 72 h were validated at the protein level (Figure 3E). Although the p21 decrease in irradiated fibroblasts in the presence of both compounds did not reach statistical significance, there was a clear declining trend, with the combination treatment presenting the best outcome (Figure 3E).

**Table 1:** List of forward and reverse primers used in RT-qPCR assays

List of primers used in RT-qPCR analysis		
*Gene/protein* name	Forward primer (5′-3′)	Reverse primer (5′-3′)
*GJA1*/Connexin43 (Cx43)	ACATGGGTGACTGGAGCGCC	ATGATCTGCAGGACCCAGAA
*HPRT1*/Hypoxanthine phosphoribosyl transferase 1 (HPRT1)	TTGAGTTTGGAAACATCTGGAG	GCCCAAAGGGAACTGATAGTC
*IL1B*/Interleukin-1 beta (IL1B)	CGAATCTCCGACCACCACTAC	TCCATGGCCACAACAACTGA
*IL6*/Interleukin 6 (IL6)	TGTAGCCGCCCCACACA	GGATGTACCGAATTTGTTTGTA
*MMP3*/Matrix metalloproteinase 3 (MMP3)	CCCTGGGTCTCTTTCACTCA	GCTGACAGCATCAAAGGACA

All analyzed genes are of human origin.

Finally, the relevance of the OLP-BIRB796 combination treatment was evaluated in a wound-healing context by scratch assay (Figure 3F). Experimentally irradiated fibroblasts were characterized by impaired *in vitro* migration, whereas 24-h treatment with 10 μM OLP or 2.5 μM BIRB796 increased their migration capacity. Notably, OLP-BIRB796 combination significantly improved *in vitro* irradiated fibroblast migration beyond the effect of either agent alone (Figure 3F), favoring a potential pro-healing effect.

### Encapsulation strategy for therapeutic delivery

The therapeutic potential of the OLP-BIRB796 combination for reversing senescence and restoring GJIC and wound healing capacity in irradiated fibroblasts was well supported by previous *in vitro* assays. However, due to their limited skin permeability and lipophilic nature, the topical delivery of these compounds required the development of a suitable nanoformulation. Lipid-based nanosystems composed of vitamin E and sphingomyelin (VSM) were selected as the delivery platform due to their biocompatibility and established performance in dermal applications.

We tested different OLP/BIRB796 loading concentrations, maintaining a 4:1 M ratio. As shown in Table 2, VSM nanosystems loaded with increasing concentrations of OLP and/or BIRB796 maintained consistent physicochemical properties: an average size around 130 nm, low polydispersity index (PDI 0.1), and near-neutral zeta potential.

**Table 2:** Physicochemical properties of blank VSM nanosystems and VSM nanosystems loaded with OLP and/or BIRB796 at different concentrations (OLP: 0–2 mg/mL; BIRB: 0.1–0.5 mg/mL) in terms of size (nm), polydispersity index, and Z-potential (mV) Mean ± SD.

VSM loading OLP/BIRB (mg/mL)	Blank VSM	0.5 OLP	1 OLP	2 OLP	0.1 BIRB	0.2 BIRB	0.5 BIRB	0.5 OLP/0.1 BIRB	1 OLP/0.2 BIRB	2 OLP/0.5 BIRB
Size (nm)	115 ± 20	110 ± 4	117 ± 4	122 ± 3	125 ± 6	128 ± 14	127 ± 2	124 ± 5	126 ± 5	130 ± 3
PDI	0.1	0.1	0.1	0.1	0.1	0.1	0.1	0.1	0.1	0.1
ζ-Potential (mV)	−4 ± 3	−7 ± 0	−9 ± 2	−8 ± 2	−3 ± 2	+2 ± 1	+4 ± 3	−2 ± 1	−1 ± 3	+3 ± 2

**Figure 4:**
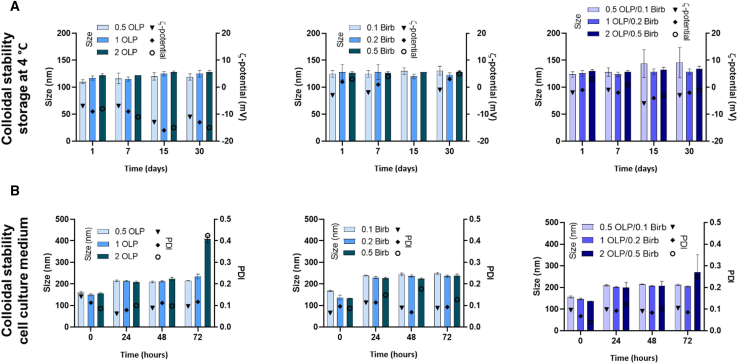
Colloidal stability of the nanosystems under storage and cell-culture conditions (A) Colloidal stability under storage at 4°C. Mean ± SD. (B) Colloidal stability during incubation in DMEM with 1% FBS for up to 72 h. Mean ± SD.

Regarding storage conditions (Figure 4A), these properties are maintained over time, up to 30 days. However, colloidal stability decreased with higher drug loading in cell culture medium (Figure 4B). Specifically, VSM systems loaded with 2 mg/mL OLP showed a size increase from 156 ± 3 to 408 ± 16 nm and a PDI increase from 0.1 to 0.4 after 72 h incubation in fetal bovine serum (FBS)-supplemented medium. A similar destabilization occurred with the 2/0.5 mg/mL OLP/BIRB796 formulation. These two conditions were therefore excluded from further studies. The optimized formulation (1/0.2 mg/mL) was used in all subsequent assays.

**Figure 5:**
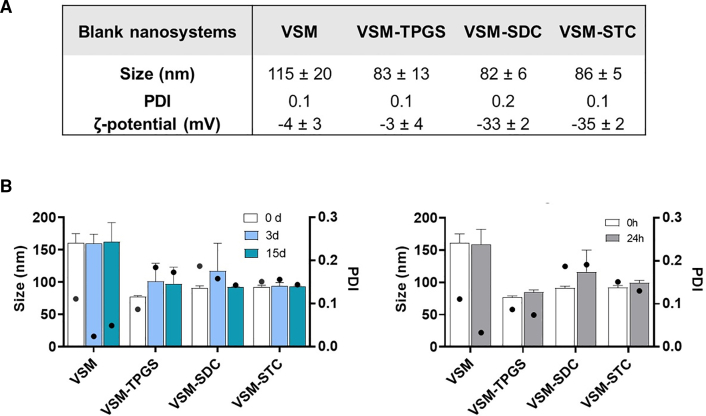
Physicochemical characteristics and stability of VSM nanosystems with OLP/BIRB796 (A) Physicochemical properties of VSM blank nanosystems. Mean ± SD. (B) Colloidal stability of VSM loaded with OLP/BIRB796 (1/0.2 mg/mL) during storage at 4°C for 15 days and at 37°C for 24 h. Mean + SD.

To optimize skin permeation, we incorporated surfactants (TPGS, SDC, and STC) into the VSM formulation. As shown in Figure 5A, the physicochemical properties of blank VSM nanosystems without drug encapsulation were first assessed. The incorporation of penetration enhancers led to a size reduction from about 115 (VSM) to 80–90 nm (with enhancers), maintaining a monodisperse profile (PDI 0.1–0.2). The zeta potential remained near neutral for VSM and VSM-TPGS but shifted to −30 mV in the SDC and STC systems due to the anionic nature of the bile salts.

In Figure 5B, drug-loaded nanosystems (OLP/BIRB796 at 1/0.2 mg/mL) were characterized in terms of colloidal stability, attending to their size and PDI. Results showed that colloidal stability remained stable at both 4°C (up to 15 days) and 37°C (24 h), simulating storage and physiological conditions, respectively. In contrast, a control solution of OLP diluted in ethanol/water at the same concentration as in the nanosystems showed visible degradation (color shift from transparent to orange) after 24 h at 37°C. This highlights the protective role of encapsulation against thermal and oxidative degradation. Although this color change was not captured photographically in the figure, it was consistently observed and supports the need for nanocarrier-mediated stabilization.

The EE was evaluated after formulation (*t* = 0 h) and after 48 h storage at 4°C. According to the results in Table 3, EE remains consistently high for both drugs in all tested formulations (>95%), confirming the robustness of the loading process and retention capacity of the nanosystems.

**Table 3:** VSM nanosystems encapsulation efficiency of OLP and BIRB796 at 1 and 0.24 mg/mL, respectively, at 0 and 48 h after storage at 4°C. Mean ± SD.

Formulation	EE OLP (%)		EE BIRB (%)	
	0 h	48 h	0 h	48 h
VSM	96 ± 6	104 ± 12	106 ± 1	109 ± 11
VSM-TPGS	96 ± 10	101 ± 3	106 ± 4	100 ± 9
VSM-SDC	110 ± 7	100 ± 4	106 ± 3	110 ± 6
VSM-STC	109 ± 5	100 ± 3	107 ± 4	97 ± 6

### Biocompatibility, cytotoxicity, and cellular internalization of VSM nanosystems in healthy and senescent fibroblasts

**Figure 6:**
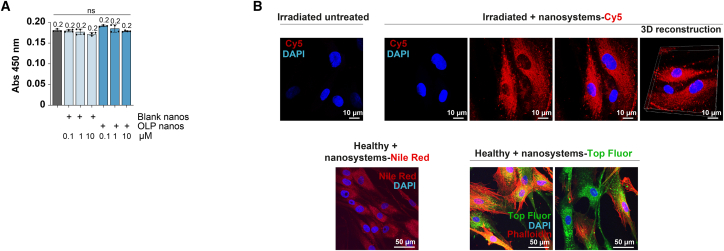
OLP-loaded VSM nanosystems do not affect cell viability and are efficiently internalized by irradiated and healthy dermal fibroblasts (A) Proliferation assay of experimentally irradiated (55 Gy) dermal fibroblasts treated for 72 h with various concentrations of OLP-loaded VSM nanosystems (0.1, 1, and 10 μM) and their corresponding blank nanoparticles. Mean ± SD, mean values specified in the graph. *n* = 3, each *n* is represented as a dot in the graph. One-way ANOVA. ns = not significant. (B) Confocal microscopy images (maximum intensity projection) and 3D reconstruction of the internalization assay of VSM nanosystems labeled with SM-Cy5 (red, top), Nile Red (red, bottom left) or SM-Top Fluor (green, bottom right), cocultured for 4 h with experimentally irradiated (55 Gy) or healthy dermal fibroblasts. Nuclei are stained with DAPI (blue), and F-actin, when corresponding, is stained with phalloidin (red). *n* = 2. Scale bars = 10, 50 μm.

Cytotoxicity of nanoparticles loaded with OLP at concentrations 0.1, 1, and 10 μM was performed in experimentally irradiated (55 Gy) dermal fibroblasts treated for 72 h and compared to that of blank nanosystems (Figure 6A) and non-encapsulated OLP (Figure 3B) at the same doses. No significant effect of nanoencapsulated OLP was detected on cellular viability at the tested concentrations (Figure 6A).

Efficient internalization of the blank VSM nanosystems by healthy and experimentally irradiated (55 Gy) dermal fibroblasts was confirmed by confocal microscopy (Figure 6B). For that, VSM nanosystems were labeled with Nile red (5 μg/mL), SM-TopFluor (4.1 μg/mL), or SM-Cy5 (2.5 μg/mL). While Nile red is expected to be encapsulated in the VSM oil core (VitE), SM-TopFluor, and SM-Cy5 would be integrated into the SM membrane of the VSM nanosystems. As it is shown in Figure 6B, the VSM nanosystems were efficiently internalized in a short period of time despite their neutral surface charge, being homogeneously distributed in the fibroblasts’ cytoplasm. Of note, the internalization ability in the irradiated fibroblasts, which present a senescent phenotype, was not impaired. VSMs capacity to be fast and efficiently internalized by other cell lines has been previously demonstrated by our research group.^30^^,^^33^^,^^37^^,^^55^

### Anti-senescent efficacy of VSM loaded with OLP-BIRB796 in irradiated fibroblasts

Having established the effects of the free compounds (Figure 3), the efficacy of the four formulated VSM nanosystems loaded with OLP/BIRB796 was evaluated. As shown in Figure S1A, senescence-associated beta-galactosidase (SA-βgal) activity, assessed by X-Gal staining (blue) in experimentally irradiated (55 Gy) dermal fibroblasts, demonstrated that OLP/BIRB796-loaded nanoparticles, particularly VSM and VSM-STC, were able to reduce radiation-induced senescence better than non-encapsulated OLP/BIRB796 combination treatment at the same dose (10 μM OLP and 2.5 μM BIRB796, 72-h treatment). The other two nanosystems, VSM-TPGS and VSM-SDC, were as efficient as the combination treatment in medium. Interestingly, blank VSM nanoparticles also showed certain anti-senescent activity (Figure S1A).

Next, preliminary protein (Figure S1B) and gene expression assays (Figure S1C) of Cx43, SASP factors (*IL1B* and *IL6*) and other senescence-related elements (*MMP3* and p21) revealed that OLP/BIRB796-loaded VSM nanosystems could similarly reduce their levels and in some cases even better than the non-encapsulated compounds, particularly VSM-STC nanosystems. As commented, blank VSM nanoparticles led to some anti-senescent effect (Figures S1B and S1C).

### OLP-BIRB796 VSM nanosystems permeability in preclinical human epidermis model

**Figure 7:**
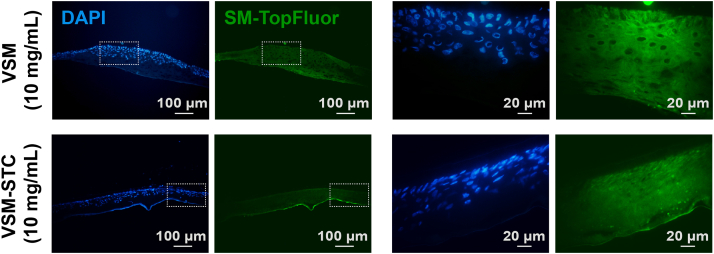
VSM and VSM-STC nanosystems penetration after 4 h treatment on an Episkin model of human epidermis VSM and VSM-STC nanosystems were incubated at a concentration of 10 mg/mL. Nuclei are stained with DAPI (blue), and nanosystems (VSM and VSM-STC) are labeled in green with SM-TopFluor. Images on the right are amplified regions of the ones on the left (dashed areas). In all pictures, the epidermal layer of keratinocytes is facing upwards. Representative images of *n* = 3. Scale bars = 20, 100 μm.

To validate the effective penetration of the VSM and VSM-STC nanosystems in a preclinical context, the EpiSkin *in vitro* model was employed. This widely recognized tool comprises a reconstructed human epidermis formed by keratinocytes cultured on a collagen matrix.^56^^,^^57^ As illustrated in Figure 7, following a 4-h treatment, both VSM and VSM-STC nanosystems labeled with SM-TopFluor were detected throughout the entire epidermal layer, forming localized depots in certain areas, demonstrating adequate skin permeability.

## Discussion

Medical ionizing radiation, including X-rays, γ-rays, and β-particles, is a prevalent therapeutic approach used for treating breast cancer and other solid tumors.^58^ However, it also unavoidably affects surrounding normal non-target tissues, particularly the skin,^13^ causing DNA damage, cell-cycle arrest, SASP production, and cellular senescence, which can ultimately impair organ function.^11^ Radiotherapy is commonly associated with adverse effects such as fibrosis, atrophy, vascular damage, chronic ulcers, and delayed or impaired wound healing.^1^^,^^59^ While transient senescence plays a beneficial role in wound healing and tissue repair,^9^^,^^60^ persistent senescent cells (common in chronic wounds induced by radiation) contribute to significant complications,^61^ highlighting the need for strategies that selectively target these cells.

Dermal fibroblasts are key players in the wound healing process by migrating to injury sites, depositing and remodeling the extracellular matrix (ECM), and facilitating wound contraction.^62^^,^^63^ Our findings show that fibroblasts isolated from the skin of breast cancer patients treated with chemotherapy and radiotherapy exhibited elevated Cx43 levels, impaired GJIC (Figure 1), increased senescence markers (Figures 3A and 3B), and heightened SASP components (*IL1B*, *IL6*, *MMP3*, and *p21*; Figure 3D), consistent with previous studies linking irradiation to increased Cx43 expression in fibroblasts^64^^,^^65^ and cardiomyocytes.^66^^,^^67^^,^^68^ The connection between Cx43 upregulation and cellular senescence is further supported by evidence of Cx43 overactivation with p53/p21-mediated senescence in osteoarthritic chondrocytes.^17^^,^^18^ Although irradiated fibroblasts display elevated Cx43 protein levels despite impaired GJIC, increased expression does not necessarily translate into enhanced gap junction or hemichannel activity. In such cases, additional mechanisms—beyond the scope of this study—may affect channel opening and molecular exchange. For example, ionizing radiation can upregulate Cx43 while inducing post-translational modifications, such as phosphorylation,^64^ which can disrupt channel trafficking or gating and thereby diminish functional GJIC.^69^

OLP has demonstrated significant antioxidant, anti-inflammatory, antimicrobial, and even antitumour properties.^70^ Treatment with OLP led to reduced Cx43 expression (Figure 2A), enhanced GJIC (Figure 2B), and decreased senescence and SASP factors (Figures 3C–3E) in fibroblasts from chemo- and radiotherapy-treated patients. These results align with previous findings showing that OLP regulates Cx43 promoter activity and suppresses NF-kB-mediated inflammatory and catabolic factors (*IL1B*, *IL6*, and *MMP3*)^18^ in osteoarthritic chondrocytes. In murine models, oral administration of OLP prevented skin damage induced by ultraviolet (UV) irradiation by inhibiting matrix metalloproteinase expression (MMP2, MMP9, and MMP13)^71^^,^^72^ and long-term OLP treatment has been shown to modulate senescence markers (β-galactosidase activity, ROS, p16, MMP2, MMP9, and IL6)^73^ and stimulate proteasome activity in fibroblasts.^70^^,^^74^ These findings underscore its therapeutic potential in skin regeneration.

Several *in vitro* and *in vivo* studies support cutaneous wound healing properties of OLP, including its effects on fibroblast and keratinocyte migration, collagen fiber deposition, and re-epithelization.^75^^,^^76^^,^^77^^,^^78^^,^^79^ In our study, OLP treatment significantly enhanced the migratory capacity of irradiated fibroblasts (Figure 3F), likely due to its effects on Cx43 downregulation and improved cellular communication (Figures 2A and 3D–3E). Certainly, lower Cx43 levels have been consistently associated with enhanced wound healing in multiple experimental models^80^^,^^81^^,^^82^^,^^83^^,^^84^^,^^85^^,^^86^^,^^87^^,^^88^ where Cx43 reduction promotes keratinocyte and fibroblast migration, reduces inflammation, and accelerates wound contraction. Indeed, persistent Cx43 upregulation has been observed in non-healing chronic wounds^89^ and its therapeutic targeting via αCT1 peptide has clearly demonstrated significant benefits in murine and porcine models, including improved scarring, mechanical properties, and faster wound closure.^90^ Clinical trials using Cx43-targeting approaches have shown promising results in treating chronic venous leg ulcers,^91^ laparoscopic surgery wounds,^92^ and diabetic foot ulcers,^93^ further supporting its relevance in wound healing therapies.

Interestingly, OLP treatment induced a phenotypic shift toward a more myofibroblast-like state in fibroblasts isolated from chemotherapy-treated skin (Figure 2B). Given that epithelial-to-mesenchymal transition (EMT) enhances fibroblast migration during wound healing,^94^ this phenotypic change, combined with Cx43 reduction (Figures 2A and 3E), likely explains the improved migration observed in irradiated fibroblasts following OLP treatment (Figure 3F).

To optimize the removal of senescent irradiated fibroblasts, we incorporated a p38 MAPK inhibitor, BIRB796, into our treatment strategy. The p38 MAPK pathway is well-established in promoting senescence and SASP production in fibroblasts,^12^^,^^52^^,^^53^ with p38 inhibition shown to reduce skin inflammation after UV B irradiation^95^ and enhance cutaneous wound healing in normal and diabetic models.^96^^,^^97^ Moreover, p38α signaling regulates MMP expression in fibroblasts,^98^^,^^99^ aligning with our observation that BIRB796 combined with OLP significantly reduced Cx43 levels, senescence, and SASP components (Figures 3C–3E) while enhancing fibroblast migration (Figure 3F). Although p38 MAPK is implicated in promoting EMT during wound healing,^94^ the removal of senescent cells achieved with BIRB796-OLP treatment appears to outweigh any potential inhibition of EMT, as indicated by the improved migratory capacity observed (Figure 3F).

The synergistic effect observed with OLP and BIRB796 likely arises from their complementary actions on converging signaling pathways that regulate both senescence and Cx43 biology. OLP reduces Cx43 gene expression and Cx43-associated senescence, including a decrease in redox stress and cytokine-driven signaling.^18^ In parallel, BIRB796 inhibits the p38 MAPK pathway, a central driver of SASP and also of Cx43 phosphorylation and turnover, which are known to impair gap junction trafficking and function.^53^^,^^100^ By simultaneously attenuating Cx43-associated senescence induction and blocking p38 MAPK-dependent post-translational modifications, the combination might produce a more robust normalization of both, Cx43 expression and channel function (GJIC), than either compound alone. OLP may primarily act upstream to reduce oxidative and inflammatory inputs that drive Cx43 transcription, whereas BIRB796 would target downstream p38-dependent phosphorylation events that impair channel trafficking and function. The translation of this dual mechanism into a more favorable phenotype, enhanced fibroblast migration and a potential improvement in wound closure capacity highlights the therapeutic promise of combined Cx43 modulation and SASP inhibition in restoring skin repair after oncological treatments.

We developed nanoformulations incorporating OLP and BIRB796 into VSM nanosystems to further enhance treatment efficacy. As previously shown by our group, VSM nanosystems are highly biocompatible with different cell lines.^30^^,^^31^^,^^32^^,^^34^^,^^35^^,^^36^^,^^37^^,^^38^ The evaluation of various OLP and BIRB796 concentrations revealed that the VSM nanosystems maintained stable physicochemical properties at an OLP concentration range from 0 to 2 mg/mL and BIRB796 concentration range from 0 to 0.5 mg/mL. Specifically, nanosystems loaded with increasing concentrations of these drugs exhibited consistent mean particle sizes (∼130 nm), low polydispersity indices (PDI ∼0.1), and neutral surface charges under initial conditions (Table 2 and Figure 4A), which are ideal characteristics for drug delivery applications. Upon incubation in cell culture medium supplemented with FBS for 24 h, a slight increase in size (from ∼156 to ∼208 nm) was observed in all drug screening concentrations, which could be related to protein adsorption and the formation of a protein corona. However, after 72 h a substantial increase in size (from ∼156 to ∼408 nm) and PDI (from 0.1 to 0.4) was observed for the nanosystems containing the highest drug concentrations, i.e., OLP/BIRB796 2/0 and 2/0.5 mg/mL, respectively (Figure 4B). These findings suggest that an increment of OLP and BIRB796 concentrations might promote aggregation or destabilization of the nanosystem structure. Similarly, nanosystems loading OLP and BIRB796 at 2 and 0.49 mg/mL, respectively, exhibited an increase in size and PDI, further emphasizing the limit of drug loading for maintaining stability. Based on these results, formulations with 1 mg/mL OLP and 0.24 mg/mL BIRB796 were selected for subsequent experiments, ensuring an optimal balance between drug loading capacity and nanosystem stability. This selection underscores the importance of tailoring drug concentrations to preserve the integrity and performance of nanosystems in biological environments.

Despite the non-ionic and anionic nature of TPGS and SDC/STC, respectively, the inclusion of these surfactants in VSM nanosystems composition could impair the biocompatibility of the nanosystems. TPGS has been shown to lead to apoptosis in different cancer cell lines but not in healthy cells,^42^ and the same trend was observed here, by maintaining the characteristic high biocompatibility of VSM nanosystems even at 500 μg/mL for 72 h. The addition of these surfactants significantly reduced the particle size of the nanosystems, particularly for those encapsulating OLP/BIRB796, which exhibited a notable decrease from 160 ± 14 nm to sizes as small as 70 ± 2 nm in the VSM-TPGS formulation (Figure 5B). This size reduction is particularly advantageous for skin applications, as smaller nanoparticles are more likely to penetrate the skin barrier effectively.^23^ Furthermore, the surface charge of the nanosystems varied according to the surfactant composition, with TPGS-containing systems maintaining a near-neutral charge and SDC/STC-containing systems acquiring a negative charge around −30 mV (Figure 5A). The latter enhances colloidal stability, which is crucial for maintaining formulation integrity during storage and application.^101^ The stability of the nanosystems was further validated under both storage (4°C for 15 and 30 days, with and without surfactants, respectively) and physiological (37°C for 24 and 72 h, with and without surfactants, respectively) conditions, demonstrating their robustness compared to the control OLP solution. The observable degradation of OLP in the control points out the necessity of nanoencapsulation to protect sensitive compounds like OLP from environmental and thermal degradation.

Moreover, the high encapsulation efficiency (∼100%) for both OLP and BIRB796, maintained even after 48 h at 4°C (Table 3), indicates the effectiveness of the VSM nanosystems in stabilizing lipophilic drugs and preventing their precipitation. Collectively, these findings reinforce the suitability of VSM nanosystems as reliable and efficient carriers for transdermal drug delivery, particularly for complex formulations involving hydrophobic and sensitive therapeutic agents.

The results regarding the cytotoxicity of nanoparticles bearing OLP demonstrate an important advantage of nanoencapsulation in ensuring safety for therapeutic applications. The absence of a significant effect on cellular viability at OLP concentrations of 0.1, 1, and 10 μM, even in experimentally irradiated (55 Gy) dermal fibroblasts treated for 72 h, highlights the biocompatibility of the nanoencapsulated formulation. This is further reinforced by the comparison with blank nanosystems and non-encapsulated OLP, where no cytotoxic differences were observed, suggesting that the encapsulation process itself does not introduce additional toxicity. These findings are particularly noteworthy given the sensitivity of irradiated fibroblasts, which are often more vulnerable to external agents due to accumulated damage and altered cellular pathways. The ability of the nanosystems to maintain cell viability at therapeutic concentrations underscores their potential as a safe delivery vehicle for OLP. Moreover, this characteristic enhances their suitability for clinical applications, particularly in oncology patients, where preserving the viability of non-target cells is critical to minimizing adverse effects during treatment.

The findings regarding the efficacy of the OLP/BIRB796-loaded VSM nanosystems provide compelling evidence of their therapeutic potential in mitigating radiation-induced cellular senescence. The superior performance of VSM and VSM-STC compared to non-encapsulated OLP/BIRB796 in reducing senescence-associated beta-galactosidase (SA-βgal) activity shows the advantages of nanoencapsulation for enhancing drug efficacy (Figure S1A). The results from protein and gene expression assays further validate these outcomes, demonstrating significant downregulation of critical senescence and SASP markers, such as Cx43, IL1B, IL6, MMP3, and p21, particularly with the VSM-STC nanosystem. This suggests that the composition, and especially the inclusion of STC, plays a pivotal role in amplifying the therapeutic effects of the encapsulated compounds (Figures S1B and S1C).

The anti-senescent activity observed in the blank VSM nanoparticles is a noteworthy finding, suggesting that the nanosystem itself may possess intrinsic properties contributing to its therapeutic potential. This effect could be linked to components such as vitamin E and SM, which are known for their antioxidative and membrane-stabilizing functions that can modulate cellular responses. Vitamin E, a well-established antioxidant, protects cells from oxidative stress, a major driver of cellular senescence. Its incorporation into nanoparticles may enhance its bioavailability and effectiveness in mitigating senescence-associated oxidative damage.^102^ Similarly, accumulating evidence highlights the role of sphingolipids, including SM, in the regulation of senescence in mammalian cells.^103^ Together, these findings underscore the dual functionality of the nanosystems: they not only improve the delivery and efficacy of encapsulated OLP and BIRB796 but also exhibit inherent anti-senescent properties that may help counteract radiation-induced cellular aging.

Compared to other similar nanoparticle-based systems, such as solid lipid nanoparticles, commonly used for transdermal delivery,^104^^,^^105^ these VSM nanosystems offer key advantages. VSM nanosystems offer smaller particle sizes and superior stability without issues of drug leakage or crystallization. Similarly, compared to polymeric nanoparticles and liposomes, VSM nanosystems provide enhanced biocompatibility due to their use of GRAS materials like Vit E and SM, making them particularly suitable for damaged or sensitive skin in oncology patients.^19^^,^^20^^,^^21^^,^^29^ Importantly, VSM nanosystems demonstrated no cytotoxicity, efficient cellular internalization in fibroblasts (Figure 6B), and encouraging results in preclinical human epidermis models (Figure 7).

The results using the EpiSkin model demonstrate the promising permeability and distribution of the VSM and VSM-STC nanosystems in a preclinical context. The presence of the nanosystems throughout the reconstructed epidermis, with localized depots after just 4 h of treatment, suggests efficient skin penetration. This is a critical finding, as it validates the suitability of these formulations for transdermal delivery, particularly for conditions requiring targeted therapeutic effects in deeper skin layers. The formation of depots also indicates potential for sustained drug release, which is advantageous for minimizing frequent application and enhancing therapeutic efficacy.

Taken together, this nanoencapsulated combination strategy offers several potential clinical advantages over conventional wound care approaches, such as standard wound moist and antimicrobial dressings, topical growth factors and conservative management. Whereas current treatments largely provide symptomatic or supportive benefit, our approach seeks to reestablish proper fibroblast function, enhancing their phenotype and restoring their capacity to communicate through gap junction channels. In addition, by simultaneously decreasing senescence and its secretory phenotype, this strategy fosters a more regenerative microenvironment to promote the capacity of tissue repair. These nanosystem-based strategies allow controlled and localized coordinated delivery of therapeutic compounds, improving their stability and bioavailability, enhancing their penetration and reducing systemic exposure in comparison with oral/systemic wound care therapies.^106^ The encapsulation strategy used here (vitamin E-sphingomyelin-based nanoemulsions) is consistent with recent reports demonstrating that sphingomyelin-containing nanoemulsions provide improved local retention and protection of labile payloads compared with free molecules. In particular, studies using analogous sphingomyelin-based nanoemulsions have shown increased retention at the site of administration and reduced early clearance versus the unencapsulated agent, supporting our choice of carrier to enhance topical stability and local bioavailability.^36^ Moreover, previous work with VSM-type formulations has documented high encapsulation efficiencies and enhanced colloidal stability of payloads,^107^ thereby rationalizing the protective effect we observe on OLP stability relative to the free compound.

In conclusion, the present study reinforces the significant potential of OLP-BIRB796 VSM nanosystems in mitigating the detrimental effects of radiotherapy-induced skin damage. We presented here the original combination of two compounds, OLP and BIRB796, targeting a novel mechanism of action for wound healing by means of senescence modulation and Cx43 tuning. Additionally, we successfully encapsulated the two compounds in combination, into VSM nanosystems, and investigated the potential of the modified formulation to enhance the therapeutic effect and penetration. OLP/BIRB796-loaded VSM nanosystems demonstrated exceptional stability, biocompatibility, and efficacy, effectively reducing radiation-induced senescence markers and SASP components.

Compared to non-encapsulated treatments, these nanosystems provided superior outcomes in senescence reduction, while maintaining safety and stability in both *in vitro* and preclinical models. Collectively, these findings position OLP/BIRB796-loaded VSM nanosystems as a promising innovative therapeutic strategy for addressing radiotherapy-associated skin complications, with potential applications in oncology and regenerative medicine. Furthermore, the dual-compound approach leveraging the synergistic effects of OLP and BIRB796 not only enhances the therapeutic potential but also opens new avenues for the development of advanced nanotherapeutics in wound healing and tissue regeneration.

The success of this study emphasizes the importance of continued research into multi-compound nanosystems, which could significantly improve clinical outcomes for patients undergoing radiotherapy and other treatments that impact skin integrity.

### Limitations of the study

It is worth noting that, given the restricted availability of patient-derived material, this work was designed as an exploratory proof-of-concept investigation and was not powered to enable breast cancer subtype-specific analyses. While our main focus was on the deleterious impact of radiotherapy and chemotherapy on dermal fibroblast senescence, GJIC impairment and wound healing capacity, it is important to acknowledge that breast tumor subtype may also influence fibroblast responses through differential exposure to systemic therapies and tumor-stromal interactions. Future studies including larger patient cohorts stratified by cancer subtype will be necessary to confirm and extend our findings in a clinically relevant context.

Other important limitations and translational challenges remain, including the need for *in vivo* validation, tumor-safety assessment and formulation optimization to address practical and regulatory challenges. Indeed, while the present work demonstrated colloidal stability of drug-loaded VSM nanosystems for up to 30 days at 4 °C and for 72 h at 37 °C, simulating storage and physiological conditions, respectively, and in agreement with other similar references concerning skin applications,^108^^,^^109^ longer-term stability assessments remain to be performed. Such studies, ideally extending beyond 30 days under controlled storage conditions and in biologically relevant media, are essential for determining the robustness and shelf-life of the formulation. Moreover, full characterization of *in vitro* release kinetics and extended retention profiles (e.g., release curves and t50 under skin-relevant conditions) are essential to translate these findings to dosing regimens. These experiments are currently being pursued in our laboratory as part of the translational development of the nanosystems.

Another meaningful aspect not addressed in the present study is the release kinetics of OLP and BIRB796 from the nanosystems. Understanding the temporal profile of drug release is crucial for predicting therapeutic timelines, bioavailability, and optimal dosing regimens. Although our focus here was on establishing proof-of-concept efficacy and physicochemical characterization, future work will include systematic release studies using skin-relevant models (e.g., dialysis assays and Franz diffusion cells) to better evaluate the controlled release properties of the nanosystems.

## Resource availability

### Lead contact

Any requests for additional information or resources should be directed to the lead contact, who will handle all inquiries, lead contact, María de la Fuente (maria.de.la.fuente.freire@sergas.es).

### Materials availability

No novel or unique reagents were generated in this study.

### Data and code availability

• All data reported in this paper is available from the lead contact upon request.
• This manuscript does not include newly developed code.
• Any further information required to support reanalysis of the data can be obtained from the lead contact upon request.

## Acknowledgments

This work was supported through funding 101079489-TWINFLAG to M.D.M. funded by Horizon Europe, Twinning (HORIZON-WIDERA-2021-ACCESS-03), EVEREST-101183034, HORIZON-MSCA-2023-SE-01 (to M.D.M.), and PDI2022-137027OB-100 to M.D.M. funded by MICIU/10.13039/501100011033AEI/10.13039/501100011033/and by FEDER/EU. M.R.-C.M. was funded with predoctoral and postdoctoral contracts FPU15/04237 and IN606B-2023/012 from 10.13039/100014440Ministerio de Ciencia, Innovación y Universidades and 10.13039/501100010801Xunta de Galicia (Spain), respectively. Authors thank the financial support given by Xunta de Galicia by Grupos de Potencial Crecemento (GPC IN607B2024/14) funded by 10.13039/501100010769Axencia Galega de Innovación (GAIN), 10.13039/501100010556Consellería de Economía, Emprego e Industria and the 10.13039/501100004587Instituto de Salud Carlos III, 10.13039/501100004587ISCIII (PI21/01262).

## Author contributions

M.R.-C.M., conceptualization, investigation, methodology, formal analysis, visualization, writing – original draft, writing – review and editing, funding acquisition; J.G.-F., conceptualization, investigation, visualization, writing – original draft, writing – review and editing; S.M.S., investigation, visualization, writing – original draft; M.A.G., investigation, methodology; S.A., investigation, methodology; A.E., investigation, methodology, visualization; M.V.-E., investigation, methodology; J.P.C., resources; B.A.N., resources; L.C.B., investigation, methodology, visualization, resources; M.D.M., conceptualization, investigation, methodology, supervision, funding acquisition, visualization writing – original draft, writing – review and editing; M.d.l.F, conceptualization, methodology, supervision, funding acquisition, writing – review and editing. All authors approved the final version of the manuscript.

## Declaration of interests

M.d.l.F. is the co-founder and CEO of DIVERSA Technologies SL. J.P.C. is the CEO and founder of ALODIA Farmacéutica. Some of the authors are inventors on a patent application related to this work (PCT/ES2020/070269).

## STAR★Methods

### Key resources table

**Table:** 

REAGENT or RESOURCE	SOURCE	IDENTIFIER
**Antibodies**		

Rabbit polyclonal anti-Cx43	Sigma-Aldrich	Cat#C6219; RRID:AB_476857
Mouse monoclonal anti-GAPDH (clone 6C5)	Invitrogen, Thermo Fisher Scientific	Cat#AM4300; RRID:AB_437392
Mouse monoclonal anti-α-Tubulin	Sigma-Aldrich	Cat#T9026; RRID:AB_477593
Rabbit monoclonal anti-p21 (clone 12D1)	Cell Signaling Technology	Cat#2947; RRID:AB_823586
Goat anti-rabbit IgG - Horseradish Peroxidase	Sigma-Aldrich	Cat#A6154; RRID:AB_258284
Sheep anti-mouse IgG - Horseradish Peroxidase	Cytiva	Cat#NA931; RRID:AB_772210
Donkey anti-rabbit IgG - Alexa Fluor 568	Invitrogen, Thermo Fisher Scientific	Cat#A10042; RRID:AB_2534017

**Biological samples**		

Untreated human primary dermal fibroblasts from the breast skin of a patient with benign pathology undergoing mammoplasty	Hospital Abente y Lago, Servizo Galego de Saúde (SERGAS)	https://xxicoruna.sergas.gal/
Untreated human primary dermal fibroblasts from the back skin of a breast cancer patient	Hospital Abente y Lago, Servizo Galego de Saúde (SERGAS)	https://xxicoruna.sergas.gal/
Radio- and chemo-therapy-treated human primary dermal fibroblasts from the breast skin of breast cancer patients	Hospital Abente y Lago, Servizo Galego de Saúde (SERGAS)	https://xxicoruna.sergas.gal/
EpiSkin™ reconstructed human epidermis	EpiSkin/L’Oréal	https://www.episkin.com/

**Chemicals, peptides, and recombinant proteins**		

Oleuropein	Cayman Chemical	Cat#21220; CAS 32619-42-4
BIRB796/Doramapimod	MedChemExpress	Cat#HY-10320; CAS 285983-48-4
F-actin/phalloidin-(Alexa Fluor 647)	Invitrogen, Thermo Fisher Scientific	Cat#A22287
Sphingomyelin (Lipoid E SM)	Lipoid GmbH	CAS 85187-10-6
Vitamin E (DL-α-tocopherol)	Sigma-Aldrich	Cat#10191-41-0
TPGS (D-α-Tocopherol Polyethylene Glycol 1000 Succinate)	Sigma-Aldrich	Cat#9002-96-4
Sodium Deoxycholate (SDC)	Sigma-Aldrich	Cat#145224-92-6
Sodium Taurocholate (STC)	Sigma-Aldrich	Cat#145-42-6; S0900000
HEC (Hydroxyethyl cellulose)	Sigma-Aldrich	Cat#9004-62-0
4′,6-Diamidino-2-phénylindole dihydrochloride (DAPI)	Sigma-Aldrich	Cat#D9542; CAS 28718-90-3
Ponceau S	Sigma-Aldrich	Cat#09189
Nile Red	Sigma-Aldrich	Cat#7385-67-3
C11 SM-TopFluor®	Avanti Polar Lipids	Cat#886209-09-2; 810265P
C16 Cyanine 5 SM	Avanti Polar Lipids	Cat#2342574-13-2; 860500C
Bromophenol blue	Sigma-Aldrich	Cat#B0126; CAS 115-39-9
Paraformaldehyde (2–4%)	Sigma-Aldrich	Cat#158127; CAS 30525-89-4
Glycerol (50% mounting medium)	Sigma-Aldrich	Cat#G5516; CAS 56-81-5
β-mercaptoethanol	Sigma-Aldrich	Cat#M6250; CAS 60-24-2
Sodium Dodecyl Sulfate (SDS)	Sigma-Aldrich	Cat#L3771; CAS 151-21-3
Tris-HCl	Sigma-Aldrich	Cat#T15760; CAS 1185-53-1
Tween 20	Sigma-Aldrich	Cat#P1379; CAS 9005-64-5
Acetonitrile (HPLC grade)	Scharlau	Cat#AC03912500
Trifluoroacetic acid (TFA)	–	–
Methanol	Scharlau	CAS [67-56-1]; EC number: 200-659-6
Absolute ethanol (EtOH, 99.7%)	VWR Chemicals	Cat#64-17-5
MilliQ water (18.2 MΩ cm)	Millipore	N/A

**Critical commercial assays**		

Cell Counting Kit-8	Dojindo Molecular Technologies	Cat#CK04
Senescence Cells Histochemical Staining Kit	Sigma-Aldrich	Cat#CS0030
TRI Reagent	Molecular Research Center	Cat#TR 118
DNase I RNase-free	Thermo Fisher Scientific	Cat#EN0521
Superscript IV VILO Master Mix	Invitrogen, Thermo Fisher Scientific	Cat#11756050
SYBR Green PowerUp	Thermo Fisher Scientific	Cat#A25741
Pierce ECL Western Blotting Substrate	Thermo Fisher Scientific	Cat#32106

**Oligonucleotides**		

Forward primer 5′-3′ *GJA1*: ACATGGGTGACTGGAGCGCC	This paper	N/A
Reverse primer 5′-3′ *GJA1: ATGATCTGCAGGACCCAGAA*	This paper	N/A
Forward primer 5′-3′ *HPRT1: TTGAGTTTGGAAACATCTGGAG*	This paper	N/A
Reverse primer 5′-3′ *HPRT1: GCCCAAAGGGAACTGATAGTC*	This paper	N/A
Forward primer 5′-3′ *IL1B*: CGAATCTCCGACCACCACTAC	This paper	N/A
Reverse primer 5′-3′ *IL1B*: TCCATGGCCACAACAACTGA	This paper	N/A
Forward primer 5′-3′ *IL6*: TGTAGCCGCCCCACACA	This paper	N/A
Reverse primer 5′-3′ *IL6*: GGATGTACCGAATTTGTTTGTA	This paper	N/A
Forward primer 5′-3′ *MMP3*: CCCTGGGTCTCTTTCACTCA	This paper	N/A
Reverse primer 5′-3′ *MMP3*: GCTGACAGCATCAAAGGACA	This paper	N/A

**Software and algorithms**		

ImageJ	NIH	https://imagej.net/ij/; RRID:SCR_003070
LaserSharp software	BioRad	N/A
GraphPad Prism software version 8.0.2	GraphPad Software, Inc.	https://www.graphpad.com/; RRID:SCR_002798
LightCycler 480 Software	Roche	N/A; RRID:SCR_012155

**Other**		

TrueBeam Linear Accelerator Radiotherapy System	Varian Medical Systems	N/A
PVDF membranes	Millipore	Cat#IPVH00010
Mowiol mounting medium	Sigma-Aldrich	Cat#81382; CAS 9002-89-5
Mini-PROTEAN Tetra electrophoresis system	Bio-Rad	Cat#1658001EDU
Mini Trans-Blot system	Bio-Rad	Cat#1703930

### Experimental model and study participant details

Cell models used in this study are human primary dermal fibroblasts isolated fresh from radio- (*n* = 1) or chemotherapy-treated (*n* = 2) breast and untreated back skin (*n* = 1) of female breast cancer patients (HER2+ and Luminal B HER2-subtypes) undergoing treatment at Hospital Abente y Lago, Servizo Galego de Saúde (SERGAS), Spain. Untreated dermal fibroblasts were also obtained from healthy breast skin of a female patient with benign pathology undergoing mammoplasty. All samples have the corresponding approval of the local ethics committee (*Comité Autonómico de Ética de la Investigación de Galicia-Autonomous Committee for Research Ethics of Galicia*, 2015/029 C.0003333) as well as the written consent of the patients.

Owing to restricted access to patient-derived material, this investigation was structured as an exploratory proof-of-concept centered on the effects of chemo- and radiotherapy regimens and did not have the statistical power necessary for subtype-specific analyses in breast cancer.

### Method details

#### Primary dermal fibroblast isolation and experimental irradiation

Skin samples were collected fresh and immediately and thoroughly washed three times in phosphate-buffered saline (PBS, MP Biomedicals) supplemented with 500 U/mL penicillin/500 μg/mL streptomycin and 1.25 μg/mL amphotericin B (Fungizone, Thermo Fisher Scientific). The hypodermic adipose layer was removed to allow proper fibroblast growth out of the dermal layer. The tissues were cut into 1 × 1 cm explants and left to adhere without medium for 15 min at 37°C to a tissue culture-treated 6-well plate or a 100 mm dish (Corning), epidermis facing up and dermis down. The bottom of the plate/dish was previously scratched with a scalpel so that tissue fragments were placed on the junctions. Skin explants were initially cultured in Dulbecco’s Modified Eagle Medium (DMEM) + 20% (v/v) fetal bovine serum (FBS) + 100 U/mL penicillin/100 μg/mL streptomycin for a few weeks until keratinocytes stopped growing and fibroblasts were noticeable; then, they were cultured in DMEM +10% (v/v) FBS +100 U/mL penicillin/100 μg/mL streptomycin and once confluent, tissue explants were removed, fibroblasts trypsinized and cultured in new vessels. Cells were cultured in a humidified incubator at 37°C and 5% CO_2_, with medium change every second or third day. Fibroblasts used in experiments were between 5 and 12 passages and Mycoplasma-free. Mycoplasma testing was routinely performed.

Dermal fibroblasts isolated from the healthy skin of a patient with benign pathology undergoing mammoplasty were experimentally irradiated with 55 Gy (Gy) using a TrueBeam Linear Accelerator Radiotherapy System (Varian Medical Systems) located at the Centro Oncológico de Galicia, Servizo Galego de Saúde (SERGAS), Spain. Conventional radiotherapy for breast cancer typically delivers daily fractions of 1.8–2 Gy, totaling 50–60 Gy over 5–6 weeks.^110^ Although *in vivo* fractionated doses are much lower per session, *in vitro* studies usually require higher single doses to induce measurable cellular responses, such as senescence, within a practical experimental time frame. In our study, the 55 Gy dose was chosen to closely approximate the cumulative biological effects observed *in vivo* upon fractionated radiotherapy, consistent with prior work employing similar single high doses to mimic the effects of fractionated radiotherapy/study the effects of senescence induction.^13^^,^^111^^,^^112^

Cells were cultured in 75 cm^2^ Nunc Easyflasks (Thermo Fisher Scientific), with at least 1 cm in thickness of culture media to allow homogeneous irradiation. For irradiation, they were placed inside a methacrylate box, with several boluses intermingled that mimic tissue properties. After radiotherapy, fibroblasts were returned to the incubator and left unperturbed for 6 h, after which their medium was renewed. Experimentally irradiated fibroblasts were left undisturbed for 4 days, and then they were subjected to various treatments and assays.

#### Drug treatments and *in vitro* assays

Primary dermal fibroblasts were treated with the polyphenol OLP and/or the SASP inhibitor BIRB796, free in the cell culture medium or encapsulated in specific nanosystems. For single 2 and 4 h OLP treatments, cells were previously serum-starved for 3 h. Long-term treatments were renewed every 48 h.

#### Immunofluorescence

2000-10000 fibroblasts were plated on glass coverslips (Thermo Fisher Scientific) previously coated with poly-D-lysine hydrobromide (Sigma-Aldrich), and cultured until they were approximately 80% confluent. They were washed with PBS, fixed with 4% paraformaldehyde (PFA, Sigma-Aldrich) in PBS for 15 min at room temperature (RT) and rinsed twice with PBS. Next, cells were incubated with 0.1 M glycine (Sigma-Aldrich) in distilled water to quench PFA for 10 min at RT, and then their membranes were permeabilized with 0.2% Triton X-100 (Sigma-Aldrich) in PBS for 10 min at RT. After two 5-min PBS washes, potential nonspecific antibody binding sites were blocked with 1% bovine serum albumin (BSA; Sigma-Aldrich) diluted in PBST (PBS+0.1% Tween 20; Sigma-Aldrich) for 30 min at RT. Cells were incubated overnight at 4°C in the dark with Cx43 primary antibody (Sigma-Aldrich, C6219), diluted in 1% BSA in PBST. The next day, cells were rinsed three times in PBS for 10 min each and then incubated for 1 h at RT in the dark with secondary antibody Rabbit IgG-Alexa Fluor 568 (Invitrogen, Thermo Fisher Scientific, A10042) diluted in 1% BSA in PBST. After that, they were washed three times with PBS for 5 min each, and their nuclei were stained with 1 μg/mL 4′,6-diamidino-2-phenylindole dihydrochloride (DAPI, Sigma-Aldrich) diluted in PBS for 10 min. Finally, cells were rinsed three times with PBS for 10 min each, and coverslips were mounted on glass microscope slides with 50% (v/v) glycerol (Sigma-Aldrich) diluted in PBS, sealing their edges with nail polish and stored at 4°C until analysis. Images were taken under an Olympus BX61 microscope coupled to a DP71 digital camera (Olympus). For quantitation purposes, regions of interest were defined with ImageJ software and their mean fluorescence intensity was calculated as the sum of the values of all the pixels within the region divided by the number of pixels, and relativized to the number of nuclei present in that area.

#### GJIC dye coupling assay

Cells were cultured until 100% confluent, and selected fibroblasts were impaled with a microelectrode (resistance 200–400 MΩ) filled with 133 mM of negatively charged (−2) 5-6-carboxyfluorescein (Sigma-Aldrich) diluted in potassium acetate 0.1 M (Sigma-Aldrich). After dye injection, hyperpolarizing pulses of 0.25–1 nA were applied. The signal emitted at 540/40 nm upon excitation at 488 nm was recorded for 10 min, using a confocal microscope MRC 1024 (BioRad) and LaserSharp software (BioRad). Three different regions of interest were defined and registered, namely the dye-injected (donor) cell, the acceptor/s cell/s that take up this gap junction-permeable dye^113^ and a background region for subtraction. Dye transfer was quantitated and expressed as the number of networks of coupled cells, the number of coupled cells per network and the coupling index, defined as the % of coupling per cell by network.

#### Protein expression analysis

2-5 million cells were harvested by trypsinization, counted and washed twice with PBS. Cell pellets for total protein isolation were thoroughly lysed on ice with a 30-gauge insulin syringe (Omnican, Braun) in ice-cold protein lysis buffer supplemented with 0.1 mM of phenylmethylsulfonyl fluoride (PMSF, Sigma-Aldrich) and 1X protease inhibitors cocktail (Sigma-Aldrich). Protein lysis buffer was composed of 150 mM NaCl (Sigma-Aldrich), 50 mM Tris-HCl (pH 7.5, Sigma-Aldrich), 5 mM EDTA (pH 8, Sigma-Aldrich), 0.1% (w/v) Sodium Dodecyl Sulfate (SDS, Sigma-Aldrich), 0.5% (v/v) Nonidet P-40 (Sigma-Aldrich) and 0.5% (v/v) N-Lauroylsarcosine (Sigma-Aldrich). Protein concentration was quantified using a Nanodrop ND-1000 (Thermo Fisher Scientific) or by Bradford assay. For western blot analysis, protein extracts were mixed with loading buffer, which consisted of 10% (v/v) β-mercaptoethanol (Sigma-Aldrich), 10% SDS, 50% (v/v) glycerol, 200 mM Tris-HCl pH 6.8 and 0.1% bromophenol blue (Sigma-Aldrich). They were boiled at 95°C for 8 min, and 20–30 μg protein was loaded into 10% acrylamide/bis-acrylamide self-made gels. Gels were run at 80 V for approximately 2 h using a Mini-PROTEAN Tetra electrophoresis system (BioRad) and then transferred to methanol-preactivated polyvinylidene fluoride (PVDF) membranes (Millipore) at 100 V and 4°C for 1–1.5 h with a Mini Trans-Blot Electrophoretic Transfer Cell (BioRad). Afterward, membranes with the transferred proteins were stained with ATX Ponceau S red staining solution (Sigma-Aldrich) for 10 min at RT, photographed in an Amersham Imager 600 (GE Healthcare) and unstained with distilled water. Membranes were blocked for 1 h at RT with 5% skim milk (Sigma-Aldrich) diluted in TBST buffer (Tris-Buffered Saline, composed of 20 mM Tris and 150 mM NaCl, supplemented with 0.05% (v/v) Tween 20, Sigma-Aldrich) with shaking. Next, membranes were incubated overnight at 4°C with rotation with primary antibodies diluted in 5% skim milk in TBST. After 4–5 TBST washes (5 min/wash), membranes were incubated for 1 h at RT with rotation with secondary antibodies conjugated to horseradish peroxidase [Rabbit IgG-HRP (Sigma-Aldrich, A6154), Mouse IgG-HRP (Sigma-Aldrich, NA-931)] diluted in 5% skim milk in TBST. Membranes were rinsed again 4-5 times (5 min/wash) with TBST, and the signal was developed with Pierce ECL Western Blotting Substrate (Thermo Fisher Scientific), in either a LAS-3000 Imager (Fujifilm) or an Amersham Imager 600 (GE Healthcare). Western blot images were analyzed and their band intensities quantified with ImageJ software. Primary antibodies used were GAPDH (Invitrogen, Thermo Fisher Scientific, AM4300), α-Tubulin (Sigma-Aldrich, T9026), Connexin43 (Sigma-Aldrich, C6219) and p21 (Cell Signaling, 2947). GAPDH, Tubulin, and Ponceau S red staining were used interchangeably as internal loading controls for quantitative analyses, depending on reagent availability at the time of each experiment.

#### RNA expression analysis

Around 1 million confluent cells were collected, their pellets thoroughly lysed with 1 mL of TRI Reagent (Molecular Research Center) on ice, and RNA was extracted according to the manufacturer’s protocol. RNA pellets were treated with DNase I RNase-free 1 U/μL (Thermo Fisher Scientific) to remove any potential remaining DNA. RNA was then quantified using a Nanodrop ND-1000 (Thermo Fisher Scientific). 1 μg RNA of each condition was retro-transcribed to complementary DNA (cDNA) with the Superscript IV VILO Master Mix (Invitrogen, Thermo Fisher Scientific) following the manufacturer’s instructions. For RT-qPCR (real-time quantitative PCR) analysis, SYBR Green PowerUp (Thermo Fisher Scientific) was used as instructed by the manufacturer, and samples were run on a LightCycler 480 System (Roche). Crossing points (CP) for each transcript were determined, relativised to those of the transcript of the reference gene *HPRT1* for each sample, and analyzed based on the delta-delta Ct method.^114^ Primers used are listed in Table 1.

#### Proliferation assay

The proliferation of primary dermal fibroblasts was evaluated using the Cell Counting Kit-8 (Dojindo Molecular Technologies). This kit comprises WST-8, a highly water-soluble tetrazolium salt, which upon reduction by cellular dehydrogenases of viable cells, gives rise to a yellow formazan dye that is soluble in cell culture media and can be quantified. Briefly, a similar number of cells (1,000–5,000) were plated in 96-well plates (Corning) and left to grow for the desired time under different treatment conditions. Then, the medium was renewed (100 μL volume/well), and 10 μL of CCK-8 reagent was added to each well. Plates were incubated in the dark for 3 h at 37°C, and absorbance was measured at 450 nm in a NanoQuant Infinite 200 plate reader (Tecan).

#### Senescence histochemical examination of SA-β gal activity

Senescence-associated beta-galactosidase activity was measured with the Senescence Cells Histochemical Staining Kit (Sigma-Aldrich) according to the manufacturer’s instructions. Cells, grown on coverslips until 70% confluence, were first washed with PBS and fixed with a buffer composed of 2% paraformaldehyde +0.2% glutaraldehyde for 7 min at RT. They were then washed thrice with PBS and incubated with a staining solution overnight at 37°C in the absence of CO_2_. This staining buffer contains X-Gal, which, upon cleavage by β-galactosidase, renders a blue product. Once the incubation time was over, cells were washed with PBS, and the coverslips were mounted on microscope slides with a 50% (v/v) glycerol solution in PBS. Cells were photographed on an Olympus BX61 microscope, and only strongly-positive blue cells were counted, expressing the data as the ratio of X-Gal positive cells relativized to the total number of cells. Cells exhibiting basal to moderate blue staining, which may also be present in healthy cells, were excluded from the analysis in order to specifically assess robust senescence induction. The classification criteria distinguishing basal-moderate from strong blue signals were established at the outset of the study and consistently applied throughout. Representative examples are provided in Figure 3A, where basal-moderate staining is observed in untreated (healthy) cells, whereas strong staining is evident under chemotherapy and radiotherapy conditions.

#### Scratch wound healing assay

To assess the capability of irradiated dermal fibroblasts to migrate, a scratch wound healing assay was performed. Cells were grown until 95% confluency and serum-starved overnight. The cellular monolayer was then scratched using a 200 μL pipette tip angled at 30°, and FBS was reduced in the medium from 10 to 0.2%. Cell migration was evaluated after 24 h-post post-scratches and expressed as the number of cells that had shifted toward the inflicted wound.

#### Preparation of blank and drug-loaded nanosystems

VSM (vitamin E–sphingomyelin) nanosystems were prepared by ethanol injection. Stock solutions of vitamin E (200 mg/mL) and sphingomyelin (40 mg/mL) were prepared in ethanol. For blank nanosystems and VSM–TPGS systems, 100 μL of the organic phase (containing VitE, SM, and TPGS when applicable) was rapidly injected into 1 mL ultrapure water at room temperature to obtain final weight ratios of 1:0.1 (VitE:SM) or 1:0.1:0.1 (VitE:SM:TPGS). For VSM–SDC and VSM–STC formulations, the same organic phase was injected into 1 mL aqueous SDC or STC (0.5 mg/mL). All preparations were gently mixed immediately after injection.

#### Nanosystem physicochemical characterization

Hydrodynamic diameter and polydispersity index (PDI) were determined by dynamic light scattering (DLS) using a Zetasizer NanoZS (Malvern Instruments). Samples were diluted 1:10 (v/v) in ultrapure water and measured at 25°C with a backscatter detection angle of 173°. ζ-potential measurements were performed after diluting samples with 700 μL of ultrapure water. Laser Doppler anemometry was used to determine electrophoretic mobility.

#### Stability assessment in biorelevant media

To examine stability under cell culture conditions, nanosystems were mixed 1:10 (v/v) with DMEM supplemented with 1% FBS and incubated at 37°C. Particle size and PDI were evaluated at selected time points by DLS as described above.

#### Preparation of fluorescently labeled nanosystems

Fluorescent VSM nanosystems were prepared using the ethanol injection method. Nile Red or SM-TopFluor (solubilized in ethanol) was added to the organic phase before injection to obtain final concentrations of 5 μg/mL and 4.1 μg/mL, respectively. For SM-Cy5 labeling, the required volume of SM-Cy5 in chloroform was placed in a microtube, the solvent was evaporated, and the remaining lipid film was combined with the other organic-phase components before injection. Final SM-Cy5 concentration in the nanosystems was 2.5 μg/mL. Nile Red partitions into the oily core of the nanosystems, whereas SM-TopFluor and SM-Cy5 incorporate into the lipid membrane.

#### Preparation of HEC gel containing VSM nanosystems

VSM-STC nanosystems were prepared as described earlier and concentrated 5.5-fold (from 1100 μL to 200 μL) using a speed vacuum concentrator at 35°C. Hydroxyethyl cellulose (HEC) was dissolved in MilliQ water for 20–30 min. Before gel formation, concentrated VSM nanosystems were added to the HEC solution at a 2:3 (v/v) ratio (HEC:nanosystems). The mixture was stirred for ∼60 min until a uniform gel formed. Final concentrations in the gel were: HEC 2% (w/v), OLP 2.2 mg/mL, BIRB796 0.48 mg/mL.

#### Encapsulation of OLP and BIRB796 in VSM nanosystems

To load drugs, OLP and/or BIRB796 prepared at 50 mg/mL in DMSO were added to the organic phase (keeping the total organic volume fixed at 100 μL). Drug molar ratio was maintained at 4:1 (OLP:BIRB796). The organic phase was injected into 1 mL aqueous phase at room temperature. For cell culture experiments, drug-loaded nanosystems were diluted in medium to 10 μM OLP and 2.5 μM BIRB796. Treatments were refreshed every 24 h. Particle size and PDI were measured as described above.

#### Preparation of drug-loaded VSM–TPGS gels

Drug-loaded VSM–TPGS nanosystems were concentrated 5.5-fold. HEC was dissolved in water at room temperature for 20 min before mixing with nanosystems at a 2:3 (v/v) ratio, yielding a final HEC concentration of 2%.

#### Measurement of drug encapsulation efficiency

Encapsulation efficiency (EE) was quantified by HPLC (Agilent 1260 Infinity II) equipped with a Poroshell 120 C18 column (250 × 4.6 mm, 5 μm). Fifty microliters of each formulation (collected from the upper phase without stirring to avoid precipitated drug) were diluted in 950 μL methanol. For OLP, 20 μL injections were eluted with acetonitrile/water (20:80, v/v) containing 0.1% acetic acid at 1 mL/min and 25°C; detection wavelength was 280 nm. For BIRB796, mobile phases (A) 0.1% TFA in water and (B) acetonitrile were used with the following gradient: 0–15 min, 80% B; 15–20 min, 80–5% B; 20–25 min, 0% B. Detection wavelength was 210 nm; flow rate 1 mL/min; column temperature 40°C; injection volume 20 μL.

#### Nanosystem internalization by human fibroblasts

Healthy or experimentally irradiated (55 Gy) dermal fibroblasts were grown on coverslips to 90% confluence. Cells were incubated for 4 h with fluorescently labeled VSM nanosystems: Nile Red (1:10 dilution), SM-TopFluor (1:50), or SM-Cy5 (1:5). Cells were washed three times with PBS, fixed with 4% paraformaldehyde for 15 min at room temperature in the dark, washed again with PBS, and counterstained with DAPI for 10 min. Coverslips were mounted with Mowiol mounting medium and stored at −20°C until imaging. Confocal imaging was performed on a Leica TCS SP5 or Nikon A1R system. Non-treated cells were processed and imaged in parallel as negative controls.

#### *In vitro* skin permeability studies

The EpiSkin reconstructed human epidermis model was used to evaluate nanosystem penetration. After topical application of VSM nanosystems for 4 h, tissues were fixed, embedded in paraffin, sectioned at 10 μm, and mounted on slides. Images were acquired with an Olympus BX61 microscope equipped with a DP71 camera. The model histologically resembles human epidermis and is widely used for permeation assays.

### Quantification and statistical analysis

Statistical analysis of data was performed using GraphPad Prism software version 8.0.2. Results are presented as mean ± standard deviation (SD). For comparing two groups, a two-tailed Student’s *t* test was carried out. Regarding the evaluation of more than two groups, multiple t-tests or one-way analysis of variance (ANOVA) were used. Statistical differences were considered significant at a *p* < 0.05, with ∗*p* < 0.05, ∗∗*p* < 0.01, ∗∗∗*p* < 0.001, ns = not significant. Most experiments were performed at least 3 independent times (*n* = 3), unless specified, generally including at least two replicates per n. All the statistical details of the experiments can be found in the figure legends.

### Additional resources

Document supplementary information. Figure S1.
