# Supplementary material for: Nanoencapsulated senotherapeutic compounds targeting connexin-43 for enhanced wound healing
Source: iScience. 2025 Dec 26;29(2):114547. doi: 10.1016/j.isci.2025.114547 (PMC12918234; doi:10.1016/j.isci.2025.114547)
Supplement: Document S1. Figure S1 [file mmc1.pdf]

iScience, Volume *29*

Supplemental information

Nanoencapsulated senotherapeutic compounds targeting connexin-43 for enhanced wound healing

Marina Rodríguez-Candela Mateos, Jenifer García-Fernández, Sofia M. Saraiva, Maria Aurora Grimaudo, Sandra Alijas, Adela Escudero, Marta Varela-Eirín, Juan Pérez Cano, Benigno Acea Nebril, Luis C. Barrio, María D. Mayan, and María de la Fuente

Supplementary Information

Nanoencapsulated Senotherapeutic Compounds Targeting Connexin-43 for Enhanced Wound Healing

Marina Rodríguez-Candela Mateos 1#, Jenifer García-Fernández 2#, Sofia M. Saraiva 2#, Maria Aurora Grimaudo 2, Sandra Alijas 2, Adela Escudero 3, Marta Varela-Eirín1, Juan Pérez Cano 4, Benigno Acea Nebril 1,5, Luis C. Barrio 3, Maria Mayan 6*, Maria de la Fuente 2,7,8*

Corresponding authors: Maria.De.La.Fuente.Freire@sergas.es and mariadolores.mayan@uvigo.gal

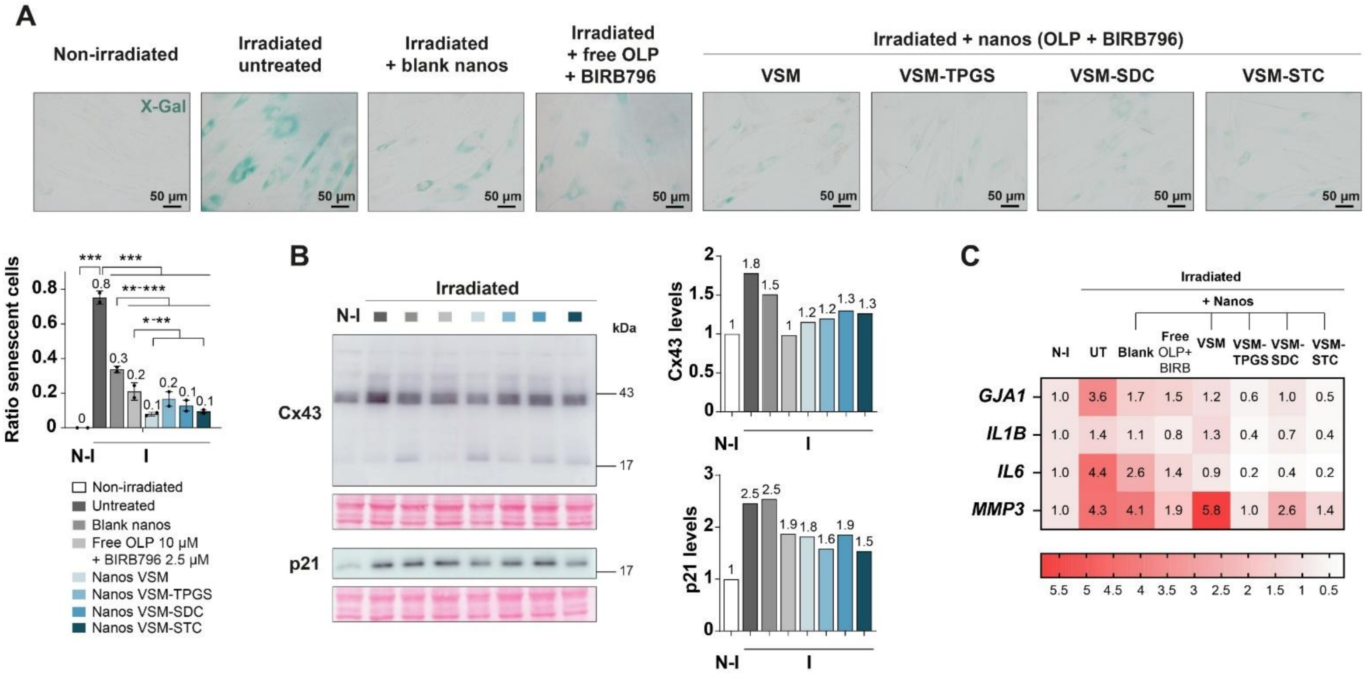

Figure Supplementary 1. OLP/BIRB796-loaded VSM nanosystems reduce overall senescence and SASP factors in experimentally-irradiated dermal fibroblasts. Non-irradiated and experimentally irradiated (55 Gy) fibroblasts were treated for 72 hours with OLP/BIRB796 either non-encapsulated (in culture medium) or in various formulations of nanosystems (VSM, VSM-TPGS, VSM-SDC, VSM-STC loaded with OLP/BIRB796), as well as with control VSM blank nanoparticles, at a final concentration of 10 µM OLP and 2.5 µM BIRB796 for all conditions. **A.** Representative images of the detection of beta-galactosidase (SA-βgal) activity by X-Gal staining (blue). Graph represents senescent cells ratio per image field analysed. Mean+SD; mean values specified in the graph. n =2 (5 images/n), each image measurement is represented as a dot in the graph. T-test. *P<0.05, **P<0.01, ***P<0.001. Scale bar = 50 µm**. B.** Representative western blots against Cx43 and p21. Ponceau S Acid Red staining is used as the total protein loading control. **C.** qPCR analysis of mRNA levels of GJA1, SASP factors (IL1B, IL6) and senescence-related component MMP3. All data were relativized to their respective HPRT-1 expression, and then to that of non-irradiated fibroblasts. Mean values are specified in the heatmap. N-I=non-irradiated. UT=untreated.
